# Both Lethal and Edema Toxins of *Bacillus anthracis* Disrupt the Human Dendritic Cell Chemokine Network

**DOI:** 10.1371/journal.pone.0043266

**Published:** 2012-08-24

**Authors:** Aurélie Cleret-Buhot, Jacques Mathieu, Jean-Nicolas Tournier, Anne Quesnel-Hellmann

**Affiliations:** Unité Interactions Hôte-Agents Pathogènes, Département de Microbiologie, Institut de Recherche Biomédicale des Armées, La Tronche, France; The Ohio State University, United States of America

## Abstract

*Bacillus anthracis,* the agent of anthrax, produces two main virulence factors: a capsule and two toxins. Both lethal toxin (LT) and edema toxin (ET) paralyze the immune defense system. Here, we analyze the effects of LT and ET on the capacity of human monocyte-derived dendritic cells (MoDC) to produce proinflammatory chemokines. We show that both toxins disrupt proinflammatory chemokine production. LT has more pronounced effects than ET on CXCL8 production, which is correlated with impaired recruitment of neutrophils *in vitro*. Finally, we show that both toxins also differentially disrupt IL-12p70, IL-10, and TNF-α production. Taken together, these results demonstrate that both *B. anthracis* toxins alter MoDC functions and the activation of the innate immune system.

## Introduction

Successful colonization of a host by pathogens relies on their capacity to evade the complex defenses created by the immune system. *Bacillus anthracis*, the agent of anthrax, has developed efficient strategies to overcome the host immune defenses, which include in particular two toxins: lethal toxin (LT) and edema toxin (ET), which are formed, respectively, by a protective antigen (PA) component associated with lethal factor (LF) or edema factor (EF) [1]. LF is a zinc-dependent metalloprotease that cleaves six of the seven mitogen-activated protein kinase (MAPK) kinases; EF is a calcium- and calmodulin-dependent adenylate cyclase that increases intracellular cAMP concentrations [2], [3]. To enter host cells, the binding moiety PA interacts with one of the two anthrax cell receptors: ANTXR1 or Tumor Endothelial Marker-8 (TEM-8) and ANTXR2 or Capillary Morphogenesis Protein-2 (CMG-2) [1]. As ANTXR1 and ANTXR2 are ubiquitously expressed, both *B. anthracis* toxins target multiple cells throughout the immune system, including polymorphonuclear neutrophils (PMN), macrophages, dendritic cells (DC), T and B cells (for review see [4]). Of importance is the fact that more innate immune cells belonging to the NK family have been recently added to the toxin target list, thereby improving our knowledge of the interactions between *B. anthracis* and the immune system [5], [6]. In addition, important information has been revealed recently by using myeloid-specific CMG2 knockout mice, this showing that impairment of myeloid cells by both toxins is critical for the establishment of the disease [7].

Among critical myeloid cells are DCs, macrophages, and neutrophils, suggesting that DCs, which stand at the crossroad of the innate and adaptive immune systems, may play a critical role throughout the disease. DCs behave classically as first-line sentinels at various entry portals throughout the body [8]. They detect microbial-associated molecular patterns (MAMP), which they integrate and process, before conveying molecular packets of information to T-cells, which is necessary for mounting an appropriate immune response. Integral to the functions of DCs is the control of their own migration, as well as the recruitment of efficient partners. The chemotactic migration of immune cells is controlled by a sophisticated communication system based on interactions of chemokines with their receptors [9]. Hence, upon MAMP recognition, DCs reprogram their chemokine receptor expression and express inflammatory chemokines, favoring the recruitment of inflammatory effectors at the site of infection. Under selective pressure, microbes have developed various strategies to subvert or escape DC responses, including the chemokine network. Dysregulating the chemokine communication system gives the pathogens the opportunity to use cell chemotaxis to their own benefit [10]. Indeed, some pathogens produce homologues of cytokines, chemokines, or their receptors to disrupt the host immune response. Infected cells can then migrate to different tissues or organs in the host where microbes can replicate and efficiently spread throughout the body.

Interestingly, we have shown in previous studies, using a mouse model of inhalational anthrax, that *B. anthracis* spores use DCs as a ‘Trojan horse’ to enter the body through a pulmonary route [11]. We, as well as others, have also demonstrated that anthrax toxins have disrupting effects on DC functions [12], [13], [14], [15].

In this study, we focus on human MoDCs infected by mutants of *Bacillus anthracis* expressing LT, ET, or both, and a non-toxinogenic mutant to evaluate the effects of LT and ET on chemokine production. We show that both LT and ET impair the production of inflammatory CXCL8, CCL2, CCL3, CCL4, and CCL5 upon infection, whereas the recruitment of PMN is significantly inhibited by LT only. Finally, we show that LT and ET also suppress cytokine production, suggesting that LT and ET have mainly anti-inflammatory effects, either affecting directly the DCs response or indirectly by the impairment of PMN recruitment.

## Materials and Methods

### Generation of MoDCs

MoDCs were generated from human peripheral blood mononuclear cells (PBMC) from healthy donors provided by the Etablissement Français du Sang (EFS). Briefly, monocytes were isolated from PBMCs of leukocyte-enriched buffy coats by negative selection, according to the manufacturer's instructions (RosetteSep, StemCell Technologies). The cells were seeded at 0.5×10^6^ cells per ml in RPMI 1640 (Sigma Aldrich), supplemented with 10% Fetal Calf Serum (Invitrogen Life Technologies), 2 mM L-glutamine (Sigma Aldrich), 100 U/ml Streptomycin, and 100 µg/ml Penicillin (both from Invitrogen Life Technologies), and containing 50 ng/ml rh GM-CSF (ProSpec-Tany TechnoGene) and 10 ng/ml rh IL-4 (PeproTech Inc). At day 7, non-adherent cells exhibited a phenotype of immature MoDCs (not shown).

### *B. anthracis* strains

The following *B. anthracis* strains provided by M. Mock (Institut Pasteur, Paris, France) were used: the parental Sterne strain 7702 (pXO1+/pXO2−), single mutant derivatives RP9 Δ*cya* and RP10 Δ*lef* that produce PA-LF and PA-EF, respectively, and double mutant RP42 Δ*lef*/Δ*cya* producing PA only.

### Infection of MoDCs

Immature MoDCs were seeded at 1.5×10^6^ cells/ml in RPMI 1640 (Sigma Aldrich) supplemented with 10% FCS (Invitrogen) and 2 mM L-glutamine (Sigma Aldrich). Cells were incubated with *B. anthracis* spores for 1 hr at a multiplicity of infection (MOI) of 20 and then washed twice and re-suspended in a medium containing 2.5 µg/ml of gentamycin (Invitrogen) to kill any remaining extracellular bacteria [16]. Culture supernatants were recovered after 18 hours. Non-infected MoDCs were used as negative controls.

### Pre-incubation of MoDCs with toxins and chemicals

MoDCs were incubated with PA (1 µg/ml) and EF and/or LF (both from List Biological Laboratories Campbell, CA, USA) at 40 ng/ml, or with 10 µM PD98059 and 10 µM SB203580 (both from Calbiochem), or with 100 µM forskolin (Sigma Aldrich), depending on the experiments, for 2 hrs at 37°C. For some experiments EF and/or LF were alternatively added at 10 and 1 ng/ml. Spores of RP42 strain (LT−/ET−) were then added at a MOI of 20 for 1 hr at 37°C. Cells were washed twice. Fresh culture medium containing gentamycin (2.5 µg/ml) was added for the remaining time.

### Chemokine assays

CCL2, CCL3, CCL4, CCL5, and CXCL8 concentrations were measured with a Bio-plex suspension array system (Bio-Rad Laboratories, France).

### Cytokine measurement in culture supernatants

IL-12, IL-10, and TNF-α secretions were measured in the supernatants of DC cultures after 18 hours using an enzyme-linked immunosorbent assay (ELISA) kit (R&D Systems, UK).

### Poly-Morphonuclear Neutrophil (PMN) migration assays

Immature MoDCs were pre-incubated with toxin when noticed and/or infected as described above. Cells were washed and incubated for 2 hours at 37°C in the lower chamber of 5 µM pore size polycarbonate filters in 24-well transwell chambers (Corning Costar). PMN isolated from blood of normal healthy donors (EFS, St Ismier, France) were then added into the upper chamber (5×10^5^ cells per well). Plates were incubated for 90 minutes at 37°C. Cells from the lower chamber were labeled with APC–conjugated antibody to CD11c, and FITC-conjugated antibody to CD66b (BD Biosciences). Cells were then analyzed on a FC500 flow cytometer (Beckman Coulter).

### Statistical analyses

Statistical analyses and graphing were performed using GraphPad Prism 5 software.

## Results

### ET and LT-secreting strains decrease the secretion of pro-inflammatory chemokines

**Figure 1:**
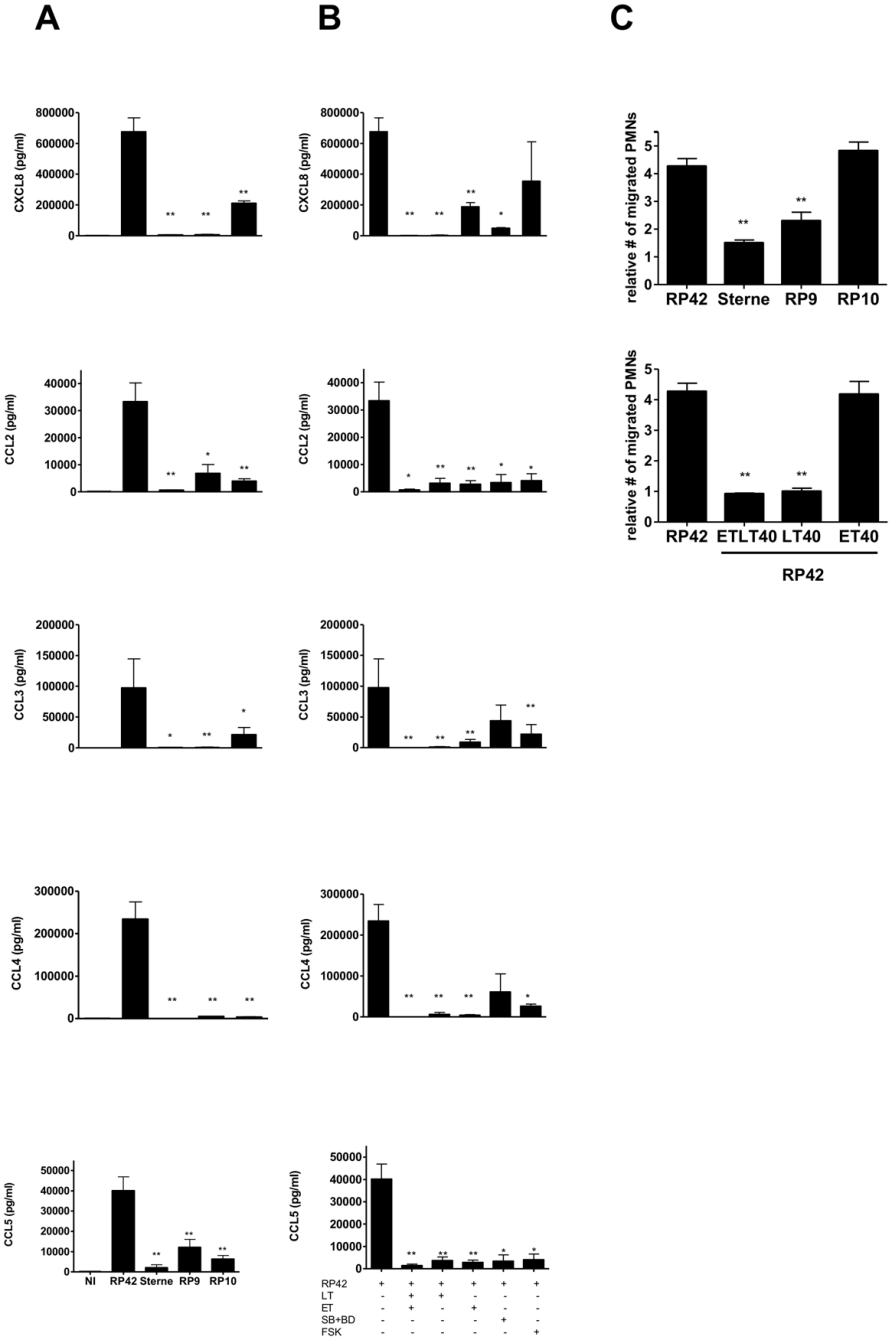
ET and LT inhibit the pro-inflammatory chemokine production, but only LT inhibits neutrophil attraction. (A) MoDCs were infected with different strains of *B. anthracis*: RP42 (LT−/ET−), Sterne (LT+/ET+), RP9 (LT+/ET−), or RP10 (LT−/ET+); (B) or pre-treated with purified toxins or forskolin, and then infected with the non-toxinogenic RP42 strain. Data show mean chemokine concentrations in culture supernatants (+/− SD) representative of three independent experiments from three different donors. (C) Human PMNs were allowed to migrate toward the supernatants of MoDCs infected with the different strains of *B. anthracis,* or pretreated with purified toxins or PD98059 and SB203580, or forskolin, and then infected with the non-toxinogenic RP42 strain. Data are presented as the number of migrated PMNs in the presence of infected MoDCs relative to control non-infected counterparts. Data are representative of three independent experiments from three different donors. Student *t-test*; *, p<0.05; **, p<0.001 as compared with RP42 infected cells. NI: not infected.

In contrast to immature MoDCs, maturing MoDCs secrete pro-inflammatory chemokines at the early stage of infection in order to coordinate the innate immune response upon recognition of MAMP [17]. Under our experimental conditions, the non-toxinogenic RP42 *B. anthracis* strain induced large amounts of monocytic chemoattractant and proinflammatory chemokine CCL2, lymphocytic chemoattractant CCL3, CCL4, CCL5, and neutrophilic chemoattractant CXCL8 (Fig. 1A). In sharp contrast, we observed that RP9 (LT-secreting strain) and RP10 (ET-secreting strain) significantly inhibited the secretion of a broad spectrum of inflammatory chemokines that included CCL2, CCL3, CCL4, CCL5, and CXCL8. Interestingly, the wild type strain secreting both LT and ET toxins inhibited secretion of all chemokines, with cooperative effects observable on CCL2 and CCL5.

To confirm the role of the toxins, cells were treated with the purified toxins and then activated with the non-toxinogenic *B. anthracis* strain RP42 (Fig. 1B). At these conditions, most chemokine secretions were almost completely inhibited, although a significant amount of CXCL8 was still produced after ET pretreatment at 40 ng/ml.

To correlate the observed effects with the molecular targets of both toxins (MAPK kinase cleavage by LT, and cAMP increase by ET), we preincubated MoDCs with PD98059 and SB203580, two inhibitors of the p38 and ERK MAPK pathways, respectively, or forskolin, an adenylyl cyclase-activating agent. Accordingly, in the presence of MAPK pathway inhibitors and forskolin, we observed similar effects to those observed in the presence of LT and ET, respectively (Fig. 1B). Taken together, these results suggest that the anthrax LT and ET disrupt chemokine production through molecular effects on MAPK pathways and dysregulation of intracellular cAMP.

### MoDCs infected with LT-secreting strains lose their capacity to attract neutrophils

Soon after MAMP recognition, MoDCs produce chemokines in order to amplify the inflammatory process and cell recruitment at the sites of infection. To evaluate the functional relevance of the aforementioned inflammatory chemokine impairment that was observed, we investigated the capacity of infected MoDCs to attract PMNs in an *in vitro* migration assay. Supernatants of human MoDCs infected with different strains of *B. anthracis* were added to the lower chamber of a transwell. Human PMNs were then added in the upper chamber and allowed to migrate for 90 minutes. As shown in Fig. 1C, MoDCs infected with the non-toxinogenic strain RP42 induced the migration of human PMNs, which can be compared to the non-infected control. MoDCs infected with the LT-expressing strains (RP9 and Sterne), but not with ET-secreting strain (RP10), showed a decreased capacity to attract PMNs. Similar effects were observed when MoDCs were treated with the purified toxins. However, even in the presence of the toxins, PMNs migrated significantly more than non-infected DCs. This suggests that ET and LT did not block all of the mechanisms involved in PMN recruitment, or that low amounts of CXCL8 are sufficient for PMN migration.

### ET and LT inhibit the secretion of cytokines by MoDCs

We next investigated whether ET and LT modulate the level of cytokines produced by MoDCs, as this has been previously shown in mouse DCs [13], [14]. We focused on three major cytokines: IL-12, the main cytokine that drives T-helper lymphocyte type 1 polarization; TNF-α, a pleiotropic cytokine with a broad range of biological activity in inflammation; and IL-10, a cytokine that down-regulates DC functions, as well as T-cell response.

**Figure 2:**
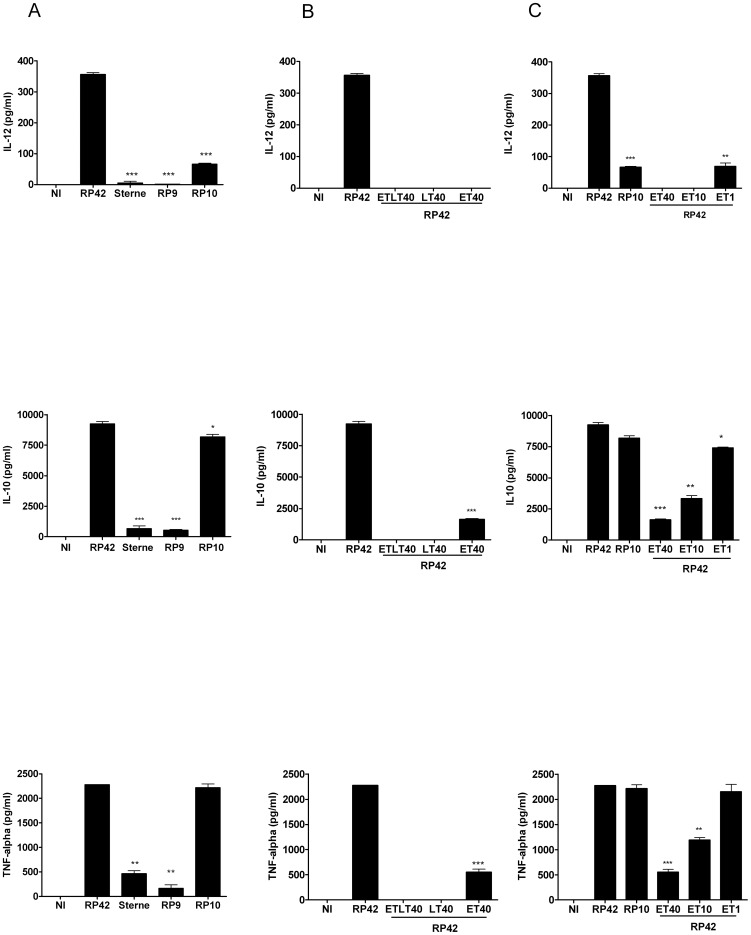
ET and LT modulate MoDCs cytokines secretions. MoDCs were infected with different strains of *B. anthracis*: RP42 (LT−/ET−), Sterne (LT+/ET+), RP9 (LT+/ET−), or RP10 (LT−/ET+) (A); or pre-treated with purified toxins, and then infected with the non-toxinogenic RP42 strain (B). Data show mean Il-12p70, IL-10, and TNF-α concentrations in culture supernatants (+/−SD) representative of three independent experiments. (C) MoDCs were pre-treated with a different range of purified ET concentrations (1, 10 and 40 ng/ml), and then infected with the non-toxinogenic RP42 strain. Data show mean Il-12p70, IL-10, and TNF-α concentrations in culture supernatants (+/−SD) representative of three independent experiments. Student *t-test*; *, p<0.05; **, p<0.001 as compared with RP42 infected cells. NI: not infected.

We observed that MoDCs produced high levels of the three cytokines when stimulated with the non-toxinogenic strain RP42. However, a dramatic decrease of IL-12 was observed in the supernatant of DCs infected with spores of ET− and/or LT-secreting strains (Fig. 2 A). Similar results were obtained when DCs were treated with the purified toxins.

IL-10 and TNF-α were significantly decreased in the presence of LT-secreting strains (Sterne and RP9) (Fig. 2A). The levels of IL-10 and TNF-α were also significantly reduced when MoDCs were pre-treated with purified LT, as compared with RP42-infected cells (Fig. 2B). However, no difference was observed when MoDC was infected with RP10, while an attenuated difference was observed with ET-pretreated cells.

To investigate the discrepancy on TNF-α and IL-10 between MoDC infected with the ET-secreting strain RP10 and the MoDC pretreated with 40 ng/ml experiments, we evaluated the effects of ET in a dose-range experiment (Fig. 2C). We observed that ET effects were dose-dependent, with a very subtle effect at the low dose of 1 ng/ml. This result suggests that the RP10 ET-secreting strain may produce very small amounts of ET upon infection.

## Discussion

In this study, we report for the first time a strong disruption of human MoDCs chemokine production by both anthrax toxins. Moreover, LT inhibited the recruitment of neutrophils by MoDCs in transwell assays. Finally, we show that ET and LT inhibited MoDCs cytokine secretion.

Our results are in contrast with those of Pickering et al. [18] which show that infection of DCs with spores of the Sterne strain induces the secretion of chemokine 7 hours after infection, as compared to non-infected cells. Moreover, Brittingham et al. [17] have reported that spores stimulated the expression of several inflammatory response genes after 2 h, followed by a striking decrease of secreted TNF-α, IL-6, and IL-8 six hours after infection with a toxinogenic strain. Taken together, these results suggest that spores of *Bacillus anthracis* are able to induce a pro-inflammatory immune response before the lethal and edema toxins become active.

The secretion of chemokines is one of the critical ways by which DCs control the immune response and the immune cell trafficking throughout the body. The chemokine-chemokine receptor network forms a redundant and complex communication network aimed at controlling monocyte, neutrophil, and lymphocyte trafficking and recruitment [9]. It was previously shown that LT can affect DC migration [15], but very little is known about the effect of LT and ET on chemokine secretion.

The inhibition of CCL2 may dampen inflammatory monocyte recruitment observed in experimental animal models of pulmonary anthrax [11]. The inhibition of CCL3, CCL4, and CCL5 may also have profound effects on the selective recruitment of Th1 and Th2 lymphocytes, and may explain the effects of toxins on the Th1/Th2 immune response [19], [20], [21]. It may also explain in part the profound alteration of the T cell response occurring in anthrax infection [22].

In addition, we also show that the complete LT-dependent inhibition of CXCL8 production by MoDCs provoked a defect in neutrophil migration. In accord with this, two independent studies on LT effects on two cellular models have described the destabilization of CXCL8 mRNA in endothelial cells [23], and prevention of histone H3 phosphorylation at Ser 10 and recruitment of p65 subunit of NF-κB at the CXCL8 promoter in epithelial cells [24]. Moreover, we have recently shown that neutrophils play a critical role in a mouse model of inhalational anthrax using a capsulated strain of *Bacillus anthracis* [25]. Taken together, these data point out various effects of LT leading to the disruption of neutrophil recruitment at the early stage of infection. This may have huge drawbacks on the subsequent events throughout the infection, as demonstrated in animal models.

The role of cytokine on human DC functions also needs to be considered. Here, we show that LT has an inhibitory effect on all three cytokines tested, while ET has only moderate effects on IL-10 and TNF-α. These results differ significantly from our previous mouse DC studies. Until now, few clues could explain species-dependent variations. Intriguingly, recent data have pointed out the effects of LF on Nlrp1, a component of the inflammasome, which bears notable differences between human and rodent (review in [26]). Moreover, very recent data have shown significant functional Nlrp1 differences within the rodent species. Rat Nlrp1 is directly cleaved by LT [27], while Nrlp1b does not appear to be a direct target in mouse macrophages [28]. This may explain why human MoDCs and mouse bone marrow-derived DCs sense their environment differently, and respond differently to LT intoxication.

Finally, we describe a novel strategy of *Bacillus anthracis* to inhibit the host immune response at the early stages of infection. ET and LT cooperate to suppress chemokine production in human dendritic cells leading to an impaired recruitment of immune cells. These novel effects of LT and ET on MoDCs functions may prove to be critical in the defense against anthrax infection.
